# Association between Concentrations of Metals in Urine and Adult Asthma: A Case-Control Study in Wuhan, China

**DOI:** 10.1371/journal.pone.0155818

**Published:** 2016-05-18

**Authors:** Xiji Huang, Jungang Xie, Xiuqing Cui, Yun Zhou, Xiaojie Wu, Wei Lu, Yan Shen, Jing Yuan, Weihong Chen

**Affiliations:** 1 Department of Occupational & Environmental Health and Key Laboratory of Environment and Health, Ministry of Education and Ministry of Environmental Protection, and State Key Laboratory of Environmental Health (Incubating), School of Public Health, Tongji Medical College, Huazhong University of Science and Technology, Wuhan, China; 2 Department of Respiratory and Critical Care Medicine, Tongji Hospital, Tongji Medical College, Huazhong University of Science and Technology, Wuhan, China; University of Cincinnati, UNITED STATES

## Abstract

**Background:**

Several metals have been reported to be associated with childhood asthma. However, the results on relationships between metals and risk of childhood asthma are inconclusive, and the research on adult asthma in the Chinese general population is rare.

**Objectives:**

To investigate potential associations between levels of urinary metals and adult asthma.

**Methods:**

A case-control study of 551 adult asthma cases and 551 gender- and age-matched controls was conducted in Wuhan, China. Demographic information was obtained, and lung function was assessed. The urinary concentrations of 22 metals were measured by inductively coupled plasma mass spectrometry.

**Results:**

After adjusting for other metalsand other covariates, urinary cadmium, molybdenum, chromium, copper, uranium and selenium were positively associated with asthma, with odds ratios (95% CI) of 1.69 (1.00, 2.85), 3.76 (2.30, 6.16), 4.89 (3.04, 7.89), 6.06 (3.27, 11.21), 6.99 (4.37, 11.19) and 9.17 (4.16, 20.21), respectively. By contrast, urinary lead, barium, iron, zinc, nickel, manganese and rubidium were negatively associated with asthma, with odds ratios (95% CI) of 0.48 (0.29, 0.80), 0.44 (0.27, 0.71), 0.41 (0.26, 0.64), 0.40 (0.24, 0.66), 0.30 (0.22, 0.41), 0.23 (0.14, 0.39) and 0.07 (0.03, 0.15), respectively. When comparing urinary metals in different subgroups of cases with those in matched controls, the associations of above 13 metals with asthma prevalence were nearly the same.

**Conclusions:**

Our results suggested that asthma prevalence in the Chinese adults was positively associated with urinary chromium, chromium, selenium, molybdenum, cadmium, and uranium, and negatively associated with urinary manganese, iron, nickel, zinc, rubidium, barium and lead. Additional research with larger populations in different regions is required to support our findings.

## Introduction

Asthma, characterized by airway hyper-responsiveness (AHR) and recurrent episodes of airway obstruction and wheezing, is one of the most common chronic inflammatory lung diseases. In recent decades, the prevalence of asthma has dramatically increased, and it affects approximately 235 million individuals worldwide [1]. In China, approximately 30 million individuals suffered from asthma, and the prevalence was 1.24% in 2011[2].

Current research has indicated that the elevated prevalence of asthma is attributable partly to increased exposures to environmental and industrial agents [3]. Along with the process of industrialization and urbanization, humans are ineluctably exposed to metals present in air, water, food, and domestic materials. Absorbed metals accumulate in tissues and organs, with half-lives ranging from several months to decades. Some metals were reported to play an important role in the expressions of inflammatory cytokines or oxidant/antioxidant balance. Since inflammatory responses and oxidative stress were the possible pathogenesis of asthma, concerns have been raised as to whether certain metals were associated with asthma prevalence [4].

In the occupational setting, metal exposure was considered as one of the primary risk factors for occupational asthma [5]. Jaakkola et al. found that the metal work was the second strongest determinant of asthma among male-dominated occupations [6]. In a case-control study in Taiwan, Wang et al. found that nonatopic occupational asthma was significantly associated with exposure to metal sensitizers and fumes [7]. Mar Ferna´ndez-Nieto also reported that chromium (Cr) and nickel (Ni) fumes could give rise to occupational asthma in exposed workers [8]. However, in the general population, levels of metal exposures are lower than in the occupational population, and studies investigating the association of asthma with metals have yielded inconsistent results. For example, Carneiro reported that low concentrations of selenium (Se) were associated with asthma [9], but a cohort study in New Zealand found no association between Se levels and asthma [10]. In addition, Ulrike Gehring reported that high levels of zinc (Zn) and iron (Fe) in ambient particulate matter were positively associated with the increased risk of asthma and allergy in schoolchildren [11], while lower concentrations of Zn and Fe in blood were observed in asthmatics [12, 13]. Hence, the exact relationships between asthma and metal exposure require more research.

With rapid process of industrialization and urbanization, China is confronted with serious metal pollution problems. Over last 30 years, the domestic emissions of cadmium (Cd), Cr, and Ni from anthropogenic sources have tripled, and the average concentrations of atmospheric arsenic (As), manganese (Mn), Cr, Ni and Cd are all beyond the limits indicated by the WHO [14]. The prevalence of asthma is relatively higher in highly industrialized cities [15]. Several epidemiological studies have focused on the dose-response association of childhood asthma with exposure to metals in China [16]. But little was known about the association between adult asthma and exposure to metals in the Chinese adult population. Adult-onset asthma is largely under investigated and far from completely understood. Compared with childhood-onset asthma, adult-onset asthma is more likely among nonatopic females and features a greater fall in lung function [17]. Moreover, previous studies have reported that source and extend of exposure, gender, age, and geography could influence the exposure levels to metals [18–20]. The role of metals in adult-onset asthma should not be extrapolated from the studies in children.

As the main route of metal excretion, urine is the preferred non-invasive matrix for metal biomonitoring, especially for surveys where large numbers of participants are involved [18]. Urinary concentrations of metals could reflect long- and/or short-term exposure to metals from all sources besides air pollution [21]. Therefore, we used the levels of metals in urine as personal metal internal exposure. In the present study, we conducted a case-control study in Wuhan, which was an industrial city and had an increasing asthma prevalence [22]. The 22 metal elements (aluminum (Al), vanadium (V), Cr, Mn, Fe, cobalt (Co), Ni, Cu, Zn, As, Se, rubidium (Rb), strontium (Sr), molybdenum (Mo), Cd, tin (Sn), antimony (Sb), barium (Ba), tungsten (W), thallium (Tl), lead (Pb), uranium (U)), part of which were reported to play some biological or pathological roles in the development of cardiopulmonary disease [23], were simultaneously determined. The objective of this study was to explore the potential associations of adult asthma with 22 urinary metals. We also compare the levels of asthma related metals in different subgroups of cases with those in the matched controls. Moreover, we investigated the correlations between urinary metals and lung function.

## Materials and Methods

### Subjects

Asthma cases were enrolled from a general hospital in Wuhan, China, from October 2010 through January 2012. All subjects were older than 18 years and lived in the communities for more than 5 years. They were outpatients and diagnosed by the qualified physicians if they had symptoms such as episodic breathlessness, wheezing, cough, and chest tightness, and/or spirometry demonstrating an increase in forced expiratory volume in 1 s (FEV_1_) of at least 12% and at least 200 ml from the prebronchodilator value according to the Global Initiative for Asthma (GINA) guidelines [24]. Adult-onset asthma has been defined as from young as 16 years [25].We excluded subjects who had any known infection, heart failure, and complications of other diseases, such as hypertension, diabetes and cerebral vessel disease at the time of study. A total of 1009 eligible adults with asthma participated in this study and the urine samples of 582 subjects had been collected. Thirty one subjects were excluded for their inadequate urine sample volume for metals assays. Thus, 551 subjects with asthma were involved in final analyses, including 312 subjects newly diagnosed as asthmatics. All subjects were subdivided into two categories by severity of asthma (intermittent, persistent) due to that small amounts of subjects had moderate persistent asthma (n = 71) or severe persistent asthma (n = 15) [24]. The numbers of subjects with intermittent and persistent asthma were 310 (56.3%) and 231 (43.7%), respectively.

Throughout the study period, we recruited the non-asthmatic controls living in the same residential areas as asthma cases for more than 5 years. A total of 3053 community residents aged 18 to 80 years agreed to participate in the study, and health examinations for the controls were performed by the same physicians. We excluded 1094 controls from this study for the history of any known infection, lung diseases, heart failure, and complications of other diseases, such as hypertension, diabetes and cerebral vessel disease. In addition, 282 controls were excluded for inadequate urine samples for metal determination. Among the remaining eligible subjects, 551 controls were matched 1:1 with asthma cases for the age (±1 year) and gender.

The study was approved by the Ethnics and Human Subject Committees of Tongji Medical School at the Huazhong University of Science and Technology (2011–17). Written informed consents were obtained from all subjects.

### Questionnaire, anthropometric measures and spirometry

Information regarding work history, educational level, occupational dust exposure, family history of asthma, tobacco smoking, physical activity, pet ownership and flower gardening was obtained by trained staff during face-to-face interviews using uniform questionnaires. For asthma cases, basic information before the diagnosis was collected. Educational level was defined as low (below senior high school), middle (senior high school to technical school), or high (college degree or higher). Family history of asthma was defined as whether the direct relatives had asthma. Work history information including job titles and beginning and ending times of jobs were collected. Occupational dust exposure was defined as self-report history of exposure to dust in the occupational setting. Individuals who had smoked more than one cigarette per day over the previous 6 months were considered as current smokers. Individuals who had drunk alcohol beverage more than once a week over the previous 6 months were considered as current drinkers. Physical activity was defined as regular exercise of more than 30 minutes in each session, at least once a week during previous 6 months.

Standing height and weight were measured without shoes before physical examinations. The body mass index (BMI) was calculated by dividing weight (kilogram) by the square of height (meter).

All subjects underwent spirometry with portable spirometers (HI-101, Chestgraphy, Japan) in sitting position with a nose clip according to the American Thoracic Society/European Respiratory Society (ATS/ERS) guidelines [26]. The spirometers were calibrated every morning before assessments according to the manufacturer’s instruction. Each subject was required to perform three satisfactory blows and the data of the best one of 3 measurements were used for analyses. The results were presented as expiratory volume (L) and percentage of the predicted values of individuals with similar characteristics (gender, age and height). FEV_1_ and forced expiratory volume in 1 s/forced vital capacity (FEV_1_/FVC) ratio were obtained.

### Determination of urinary metals and creatinine

On the morning of recruitment days, spot urine samples were collected, divided into aliquots, and subsequently stored in polyethylene tubes at -20°C until further processing. We determined concentrations of 22 metals using inductively coupled plasma mass spectrometry (ICP-MS) with an Agilent 7700x instrument (Agilent Technologies, Waldbronn, Germany). We followed the previously reported method with minor modifications [27]. Standard reference material (SRM) 2670a (Toxic Elements in Urine) containing low and high concentration levels and 1640a (Trace Elements in Natural Water) purchased from NIST (National Institute of Standards and Technology, Gaithersburg, Maryland, USA) were used for quality control. Accuracy was estimated by comparing the difference between the certified values available and the measured values with their uncertainty according to the calculation method reported elsewhere [28]. The results measured by this method were in line with the SRM 2670a certified values. SRM 1640a was used to monitor the accuracy of procedure for each analytic batch. The mean of three replicate measurements for every metal was reported. The limits of quantification (LOQ) for 22 metals ranged from 0.0004 to 0.3934 μg/L. For metal concentrations below the LOQ, we assigned half the LOQ for the calculation. We measured urinary creatinine concentrations using a fully automated clinical chemistry analyzer (Mindray Medical International Ltd., Shenzhen, China). The concentrations of metals were adjusted by creatinine and were presented as micrograms per gram of creatinine.

### Statistical analysis

Chi-square test or Student’s t-test was used to compare basic characteristics between asthma cases and controls as appropriate. We compared the urinary concentrations of the 22 metals between the two groups using a Wilcoxon signed-rank test. The correlations among the 22 metals were analyzed by using Spearman’s rank correlation analysis. To improve the normalization of concentrations of metals, we transformed them by natural logarithm transformation [29]. We used conditional logistic regression models to analyze relationships between individual metals and asthma after adjusting for asthma-related factors such as educational level, occupational dust exposure, family history of asthma, tobacco smoking, pet ownership, flower gardening, physical activity and BMI. Due to multicollinearity, we subsequently constructed logistic regression models including all metals related to asthma in single-metal models and the above confounders and used a backward elimination procedure to retain the metals that predicted the outcome at p<0.05. The correlation analyses between urinary concentrations of asthma-related metals in multiple-metal models and lung function were also performed by the partial Spearman’s correlation analysis with adjustment for age, gender, occupational dust exposure, family history of asthma, tobacco smoking, physical activity, and BMI. In addition, we used the false discovery rate (FDR) to adjust *p* values for multiple testing [30]. The level of significance was set at p<0.05. We performed statistical analyses using SAS software (version 9.1; SAS Institute Inc., USA).

## Results

### General characteristics

A total of 551 asthma cases and 551 matched non-asthmatic controls were included in the analysis. The participants aged ranged from 18 to 78 years, and mean age was 42.41±12.80 years for the asthma cases and 42.74±12.56 years for controls. The age at diagnosis of asthma for cases varied from 16 to 72 years. Table 1 presents the characteristics, lifestyle and clinical features of cases and controls. Compared with controls, the percentage of asthma cases with occupational dust exposure or family history of asthma was significantly higher (p<0.05, p = 0.0001, respectively). The distributions of tobacco smoking were significantly different between the two groups (p = 0.0001). Pet ownership was more prevalent among the asthma cases than among the controls (p<0.0011). The mean FEV_1_% (88.07±13.76) and FEV_1_/FVC ratio (87.90±8.80) for the controls were significantly higher than those for asthma cases (82.85±24.00 and 71.40±14.09, respectively) (both p<0.0001). The mean BMI of the asthma cases (23.13±4.21) was lower than that of the controls (24.00±3.66) (p = 0.0002).

**Table 1:** Demographic characteristics, lifestyle and clinical features of asthma cases and controls.

Characteristic	Control (*n* = 551)	Case (*n* = 551)	p-value^a^
Male, n (%)	237 (43.01)	237 (43.01)	
Age (years, Mean±SD)	42.74±12.56	42.41±12.80	0.3769
Educational level, n (%)			0.0005
Low	260 (47.19)	308 (55.90)	
Middle	197 (35.75)	138 (25.05)	
High	94 (17.06)	105 (19.06)	
Exposure to dust, n (%)	69 (12.52)	96 (17.42)	0.0224
Family history of asthma, n (%)	11 (2.00)	62 (11.25)	0.0001
Tobacco smoking, n (%)			<0.0001
Nonsmokers	392 (71.14)	434(78.77)	
Former smokers	27 (4.90)	42(7.62)	
Current smokers	132 (23.96)	75(13.61)	
Physical activity, n (%)	188 (34.12)	126 (22.87)	0.0001
Pet ownership, n (%)	56 (10.16)	93 (16.88)	0.0011
Flower gardening, n (%)	169 (30.67)	108 (19.60)	<0.0001
BMI level, n (%)			<0.0001
Underweight, <18.5 kg/m^2^	22 (3.99)	48 (8.71)	
Normal weight, 18.5–24 kg/m^2^	267 (48.46)	309 (56.08)	
Overweight, 24–28 kg/m^2^	198 (35.93)	151 (27.40)	
Obese, ≥28 kg/m^2^	64 (11.62)	43 (7.80)	
BMI (kg/m^2^, Mean±SD)	24.00±3.66	23.13±4.21	0.0002
Spirometric indices (Mean±SD)			
FEV_1_ (L)	2.54±0.64	2.33±0.84	<0.0001
FEV_1_(% predicted)	88.07±13.76	82.85±24.00	<0.0001
FEV_1_/FVC (%)	87.90±8.80	71.40±14.09	<0.0001

Abbreviation: SD, standard deviation; BMI, body mass index; FEV_1_, forced expiratory volume in 1 s; FVC, forced vital capacity.

^a^Student’s t-test for continuous variables and Chi-square test for categorical variables.

### Urinary metal concentrations

Table 2 compares the creatinine-adjusted urinary metal concentrations (μg/g creatinine) between asthma cases and controls. The urinary concentrations of Sn, Ni, V, Pb and W were < LOQ in 26.50%, 15.97%, 8.35%, 5.17% and 2.09% of the samples, respectively. Less than 1% of the samples were < LOQ for Cr, Mn, Fe, Co, Cu, Sb and U. The geometric mean urinary concentrations of eleven metals (Cr, Cu, As, Se, Sr, Mo, Cd, Sn, Sb, W and U) were significantly higher in the asthma cases than in the controls (p<0.01). In contrast, the geometric mean urinary concentrations of ten metals (Al, V, Mn, Fe, Ni, Zn, Rb, Ba, Tl and Pb) were significantly lower in the asthma cases (p<0.05). We observed no significant differences in urinary Co levels between the two groups (p = 0.872).

**Table 2:** Comparison of metal concentrations (μg/g creatinine) in urine [geometric means (25th, 75th percentiles)] between asthma cases and controls.

Metal	Control (*n* = 551)	Case (*n* = 551)	p-value	n (%)<LOQ	Reference values^a^
**Al**	21.54 (12.53, 33.91)	14.73 (7.55, 25.10)	<0.0001	0(0.00)	
**V**	0.33 (0.20, 0.52)	0.27 (0.20, 0.97)	<0.0001	92(8.35)	
**Cr**	0.94 (0.53, 1.52)	1.52 (0.81, 2.77)	<0.0001	1(0.09)	
**Mn**	1.48 (0.88, 2.42)	0.58 (0.27, 1.21)	<0.0001	7(0.64)	
**Fe**	47.94 (25.66, 85.24)	14.15 (6.99, 26.12)	<0.0001	5(0.45)	
**Co**	0.18 (0.10, 0.34)	0.15 (0.10, 0.31)	0.8720	2(0.18)	0.29 (0.26, 0.31)
**Ni**	1.45 (0.87, 2.37)	0.38 (0.10, 1.31)	<0.0001	176(15.97)	
**Cu**	4.71 (3.13, 6.27)	5.81 (3.69, 8.35)	<0.0001	0(0.00)	
**Zn**	165.67 (118.40, 248.60)	115.58 (73.46, 180.60)	<0.0001	0(0.00)	
**As**	16.44 (11.18, 22.87)	19.11 (12.79, 24.90)	<0.0001	0(0.00)	8.30 (7.19, 9.57)
**Se**	4.81 (3.34, 6.75)	7.69 (5.58, 10.67)	<0.0001	0(0.00)	
**Rb**	1187.97 (853.76, 1747.47)	1074.92 (775.47, 1501.06)	0.0002	0(0.00)	
**Sr**	77.48 (51.74, 122.10)	93.69 (56.19, 153.2)	<0.0001	0(0.00)	
**Mo**	27.11 (18.01, 40.89)	42.95 (26.51, 66.03)	<0.0001	0(0.00)	35.90 (34.00, 38.00)
**Cd**	0.49 (0.31, 0.76)	0.62 (0.40, 0.90)	<0.0001	0(0.00)	0.26 (0.24, 0.28)
**Sn**	0.17 (0.11, 0.27)	0.20 (0.12, 0.33)	0.0018	292(26.50)	
**Sb**	0.10 (0.07, 0.14)	0.11 (0.07, 0.17)	0.0026	1(0.09)	
**Ba**	2.56 (1.49, 4.13)	1.82 (0.95, 3.07)	<0.0001	0(0.00)	1.34 (1.19, 1.56)
**W**	0.08 (0.05, 0.14)	0.11 (0.06, 0.19)	<0.0001	23(2.09)	
**Tl**	0.36 (0.25, 0.55)	0.32 (0.21, 0.48)	<0.0001	0(0.00)	0.15 (0.13, 0.16)
**Pb**	1.93 (1.25, 3.01)	1.39 (0.90, 2.05)	<0.0001	57(5.17)	0.63 (0.58, 0.67)
**U**	0.02 (0.01, 0.03)	0.02 (0.01, 0.04)	0.0019	5(0.45)	

Abbreviation: LOQ, limits of quantification.

^a^The values were from *The Fourth National Report on Human Exposure to Environmental Chemicals* (U.S. 2009) for 20 years and older.

Correlation analysis showed that the 22 metals were significantly correlated with one another except for Mn vs. Se (p = 0.34), Mn vs. Mo (p = 0.53), Fe vs. Mo (p = 0.39), Fe vs. W (p = 0.23) and Ni vs. Mo (p = 0.08) (Table 3).

**Table 3:** Correlation coefficients of urinary metals (μg/g creatinine) in the entire study population.^a^

	Al	V	Cr	Mn	Fe	Co	Ni	Cu	Zn	As	Se	Rb	Sr	Mo	Cd	Sn	Sb	Ba	W	Tl	Pb	U
**Al**	1.00																					
**V**	0.43	1.00																				
**Cr**	0.43	0.69	1.00																			
**Mn**	0.73	0.12	0.35	1.00																		
**Fe**	0.57	0.07	0.21	0.73	1.00																	
**Co**	0.38	0.31	0.37	0.39	0.33	1.00																
**Ni**	0.47	0.27	0.13	0.46	0.48	0.47	1.00															
**Cu**	0.46	0.34	0.64	0.48	0.35	0.41	0.26	1.00														
**Zn**	0.38	0.09	0.14	0.46	0.46	0.23	0.41	0.42	1.00													
**As**	0.14	0.30	0.21	0.09	0.09	0.31	0.11	0.24	0.22	1.00												
**Se**	0.13	0.38	0.53	0.03	-0.06	0.31	-0.1	0.39	0.14	0.53	1.00											
**Rb**	0.22	0.22	0.21	0.26	0.23	0.31	0.16	0.25	0.18	0.47	0.41	1.00										
**Sr**	0.32	0.36	0.38	0.23	0.17	0.38	0.16	0.33	0.24	0.37	0.39	0.16	1.00									
**Mo**	0.08	0.26	0.25	0.02	-0.03	0.34	0.05	0.28	0.11	0.52	0.47	0.28	0.38	1.00								
**Cd**	0.21	0.20	0.23	0.13	0.09	0.41	0.14	0.39	0.23	0.48	0.45	0.43	0.34	0.43	1.00							
**Sn**	0.39	0.23	0.27	0.28	0.22	0.30	0.18	0.32	0.27	0.28	0.34	0.29	0.38	0.30	0.37	1.00						
**Sb**	0.44	0.31	0.29	0.33	0.24	0.36	0.25	0.44	0.32	0.42	0.35	0.37	0.46	0.42	0.46	0.55	1.00					
**Ba**	0.62	0.28	0.34	0.58	0.47	0.34	0.38	0.39	0.35	0.15	0.08	0.13	0.63	0.11	0.22	0.39	0.50	1.00				
**W**	0.23	0.27	0.18	0.09	0.04	0.25	0.14	0.19	0.14	0.32	0.30	0.19	0.36	0.40	0.26	0.39	0.53	0.27	1.00			
**Tl**	0.32	0.24	0.27	0.33	0.29	0.39	0.21	0.29	0.22	0.38	0.32	0.75	0.33	0.28	0.40	0.36	0.40	0.33	0.22	1.00		
**Pb**	0.54	0.25	0.30	0.54	0.47	0.35	0.37	0.35	0.42	0.25	0.20	0.35	0.47	0.21	0.33	0.46	0.55	0.61	0.32	0.49	1.00	
**U**	0.41	0.18	0.14	0.27	0.18	0.21	0.20	0.33	0.25	0.23	0.10	0.22	0.37	0.28	0.34	0.48	0.71	0.49	0.46	0.31	0.45	1.00

^a^ p<0.05 for the correlations between all the metals, except for Mn vs. Se (p = 0.34), Mn vs. Mo (p = 0.53), Fe vs. Mo (p = 0.39), Fe vs. W (p = 0.23) and Ni vs. Mo (p = 0.08).

### Associations between urinary metals and asthma

**Fig 1:**
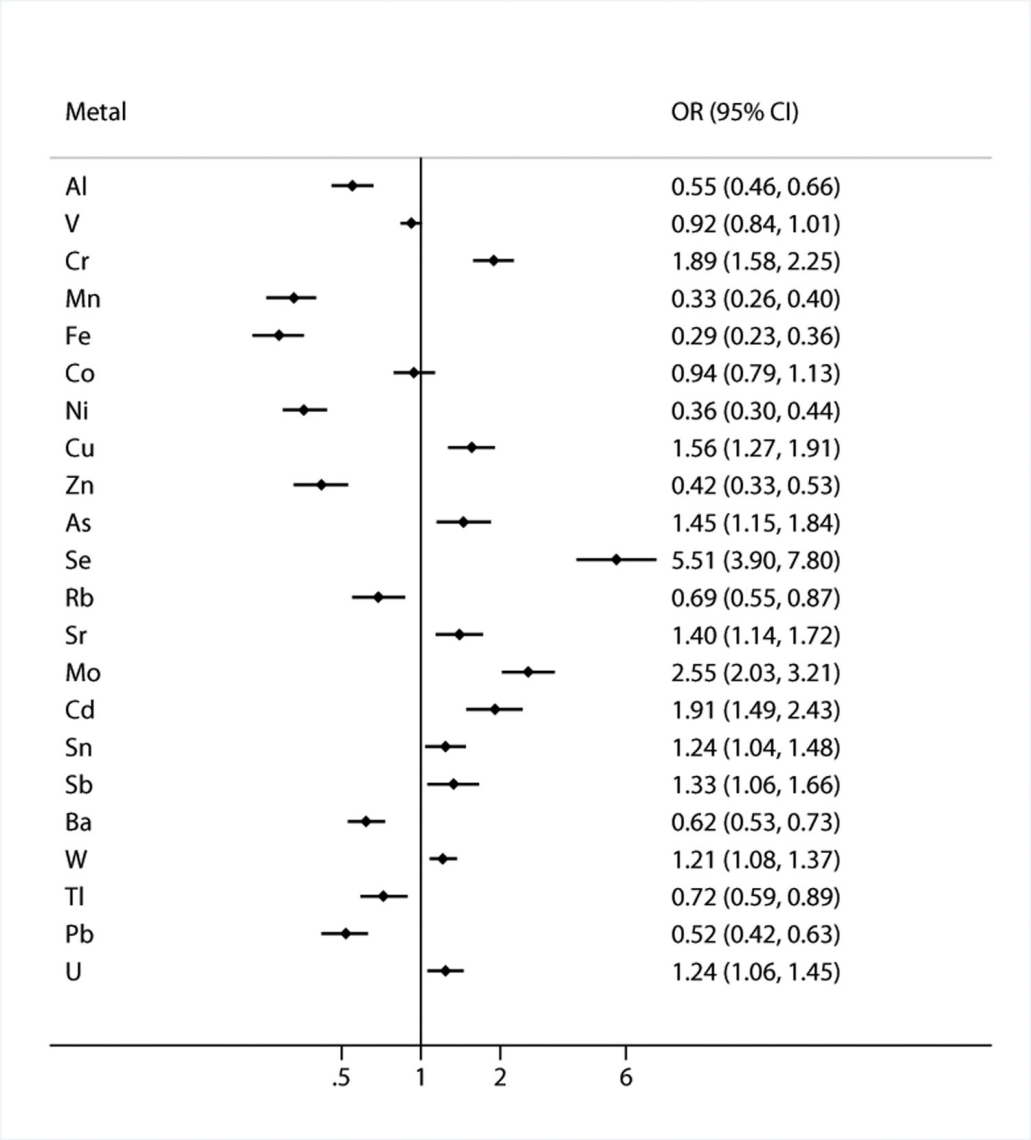
Adjusted odds ratio and 95% confidential interval for asthma by considering urinary concentrations of metals as continuous variables. The models were adjusted for educational level, occupational dust exposure, family history of asthma, tobacco smoking, pet ownership, flower gardening, physical activity, and body mass index.

When considered as continuous variables, 20 metals were associated with asthma (Fig 1). The elevated urinary levels of eleven metals (Cr, Cu, As, Se, Sr, Mo, Cd, Sn, Sb, W and U) were significantly positively associated with the prevalence of asthma, with odds ratios (ORs) ranging from 1.21 to 5.51. Furthermore, we observed significantly negative associations between nine metals (Al, Mn, Fe, Ni, Zn, Rb, Ba, Tl and Pb) and asthma, with ORs ranging from 0.29 to 0.72. We found no associations of V and Co with asthma. When metal concentrations were divided into quartiles, the associations between the above mentioned 20 metals and asthma were unchanged, except that for Sn (Table 4). Compared with the lowest quartiles, the ORs (95% confidential interval (CI)) for the highest quartiles of urinary Se and Mo were 11.40 (6.82, 19.05) and 5.67 (3.63, 8.85), respectively. The ORs of the highest quartiles of urinary Mn, Fe and Ni were less than 0.10 when compared with the lowest quartiles. In all, through single-metal models, we observed that 19 metals were significantly associated with asthma. We got similar results when analyzing the associations of asthma with single metal element by excluding the subjects whose concentration of the metal element with metal levels was below the limit of detection.

**Table 4:** Adjusted odds ratio and 95% confidential interval for asthma by considering urinary concentrations of metals as categorical variables.

Metal	quartile 1	quartile 2	quartile 3	quartile 4	p-value for trend	p-value for trend ^a^
**Al**	1 (ref)	0.41 (0.27, 0.64)	0.24 (0.16, 0.38)	0.23 (0.15, 0.35)	<0.0001	<0.0001
**V**	1 (ref)	0.35 (0.23, 0.54)	0.92 (0.62, 1.38)	2.64 (1.72, 4.05)	<0.0001	<0.0001
**Cr**	1 (ref)	1.52 (1.02, 2.28)	2.08 (1.39, 3.13)	4.60 (2.98, 7.09)	<0.0001	<0.0001
**Mn**	1 (ref)	0.11 (0.06, 0.22)	0.04 (0.02, 0.08)	0.03 (0.02, 0.06)	<0.0001	<0.0001
**Fe**	1 (ref)	0.10 (0.05, 0.21)	0.03 (0.01, 0.06)	0.02 (0.01, 0.04)	<0.0001	<0.0001
**Co**	1 (ref)	1.18 (0.79, 1.77)	1.20 (0.79, 1.84)	0.90 (0.57, 1.42)	0.6564	0.6564
**Ni**	1 (ref)	0.04 (0.02, 0.08)	0.03 (0.01, 0.06)	0.02 (0.01, 0.05)	<0.0001	<0.0001
**Cu**	1 (ref)	1.27 (0.85, 1.92)	1.49 (1.00, 2.23)	2.17 (1.47, 3.19)	<0.0001	<0.0001
**Zn**	1 (ref)	0.45 (0.29, 0.69)	0.21 (0.14, 0.33)	0.19 (0.12, 0.29)	<0.0001	<0.0001
**As**	1 (ref)	1.61 (1.09, 2.38)	1.61 (1.10, 2.38)	1.78 (1.19, 2.65)	0.0072	0.0088
**Se**	1 (ref)	1.93 (1.24, 3.00)	4.27 (2.70, 6.74)	11.40 (6.82, 19.05)	<0.0001	<0.0001
**Rb**	1 (ref)	0.90 (0.62, 1.33)	0.65 (0.44, 0.96)	0.49 (0.33, 0.73)	0.0002	0.0003
**Sr**	1 (ref)	1.00 (0.67, 1.48)	1.12 (0.75, 1.66)	1.85 (1.23, 2.77)	0.0022	0.0028
**Mo**	1 (ref)	1.53 (1.02, 2.30)	2.65 (1.76, 3.99)	5.67 (3.63, 8.85)	<0.0001	<0.0001
**Cd**	1 (ref)	2.06 (1.37, 3.11)	2.42 (1.56, 3.75)	3.05 (1.94, 4.81)	<0.0001	<0.0001
**Sn**	1 (ref)	1.04 (0.70, 1.55)	1.15 (0.77, 1.72)	1.39 (0.94, 2.05)	0.0831	0.0871
**Sb**	1 (ref)	0.65 (0.43, 0.98)	1.06 (0.71, 1.59)	1.40 (0.94, 2.08)	0.0144	0.0167
**Ba**	1 (ref)	0.51 (0.34, 0.76)	0.40 (0.27, 0.61)	0.27 (0.18, 0.41)	<0.0001	<0.0001
**W**	1 (ref)	1.33 (0.90, 1.97)	2.14 (1.43, 3.18)	2.46 (1.65, 3.67)	<0.0001	<0.0001
**Tl**	1 (ref)	1.01 (0.68, 1.48)	0.70 (0.47, 1.05)	0.55 (0.37, 0.80)	0.0004	0.0006
**Pb**	1 (ref)	0.58 (0.38, 0.89)	0.35 (0.22, 0.54)	0.19 (0.12, 0.29)	<0.0001	<0.0001
**U**	1 (ref)	0.68 (0.46, 1.02)	0.77 (0.52, 1.14)	1.49 (1.00, 2.21)	0.0237	0.0261

The model was adjusted by educational level, occupational dust exposure, family history of asthma, tobacco smoking, pet ownership, flower gardening, physical activity, and body mass index.

^a^ p-value was adjusted by false discovery rate (FDR) for multiple testing.

**Fig 2:**
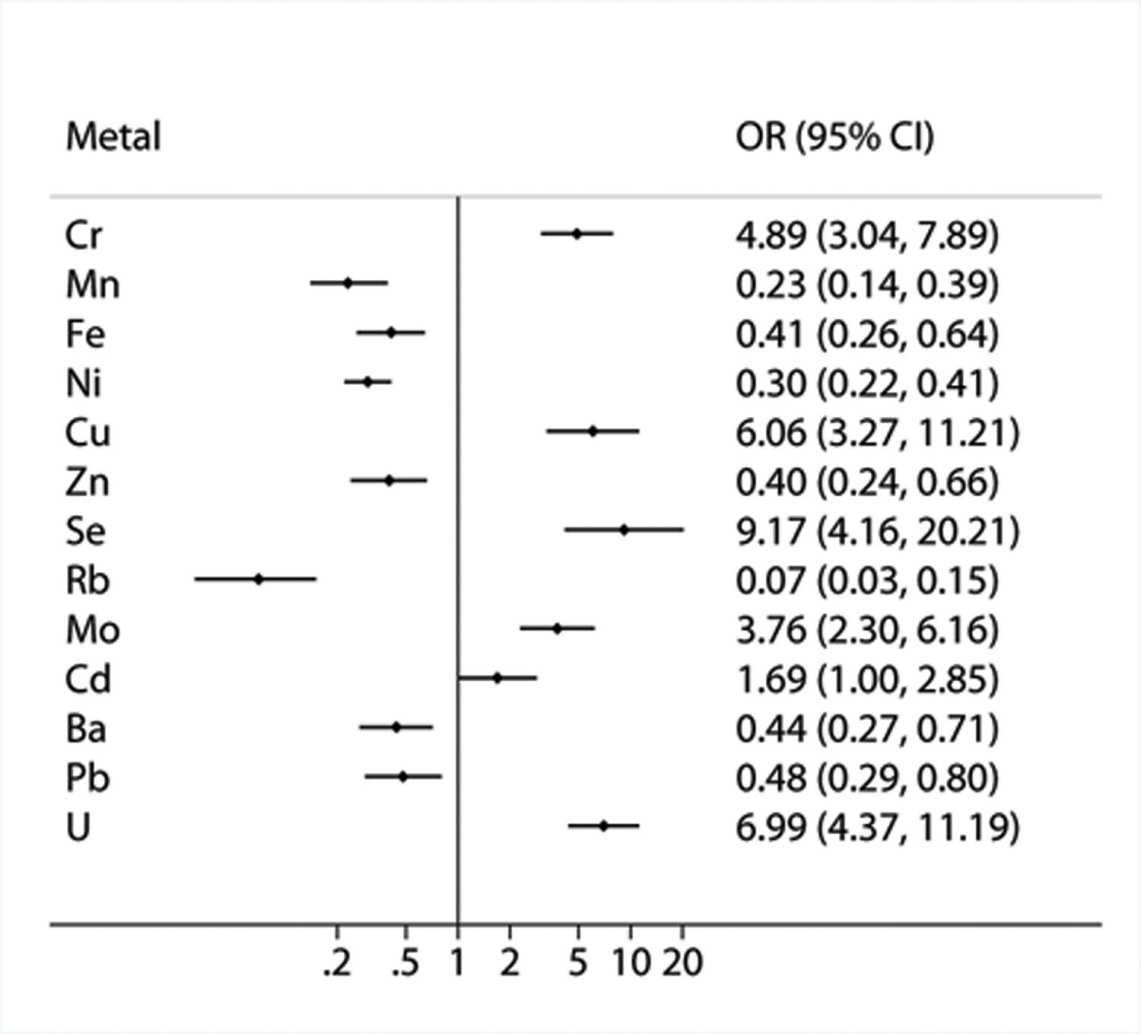
Associations between urinary metals and asthma based on the multiple-metal models. Metals were selected among 19 asthma-related metals from single-metal models by backward elimination in the multivariate logistic regression model (alpha = 0.05) with adjustment for age, gender, tobacco smoking, educational level, occupational dust exposure, family history of asthma, pet ownership, flower gardening, physical activity and body mass index. Abbreviation: OR: odds ratio; CI: confidential interval.

We further constructed multiple-metal model analyses including the above 19 metals using backward elimination (Fig 2). Six metals (Cd, Mo, Cr, Cu, U and Se) were significantly positively associated with the prevalence of asthma (OR (95%CI): 1.69 (1.00, 2.85) for Cd; 3.76 (2.30, 6.16) for Mo; 4.89 (3.04, 7.89) for Cr; 6.06 (3.27, 11.21) for Cu; 6.99 (4.37, 11.19) for U; 9.17 (4.16, 20.21) for Se). In contrast, elevated levels of seven metals (Pb, Ba, Fe, Zn, Ni, Mn and Rb) in urine were significantly negatively associated with the prevalence of asthma (OR (95%CI): 0.48 (0.29, 0.80) for Pb; 0.44 (0.27, 0.71) for Ba; 0.41(0.26, 0.64) for Fe; 0.40 (0.24, 0.66) for Zn; 0.30 (0.22, 0.41) for Ni; 0.23 (0.14, 0.39) for Mn; 0.07 (0.03, 0.15) for Rb).

**Fig 3:**
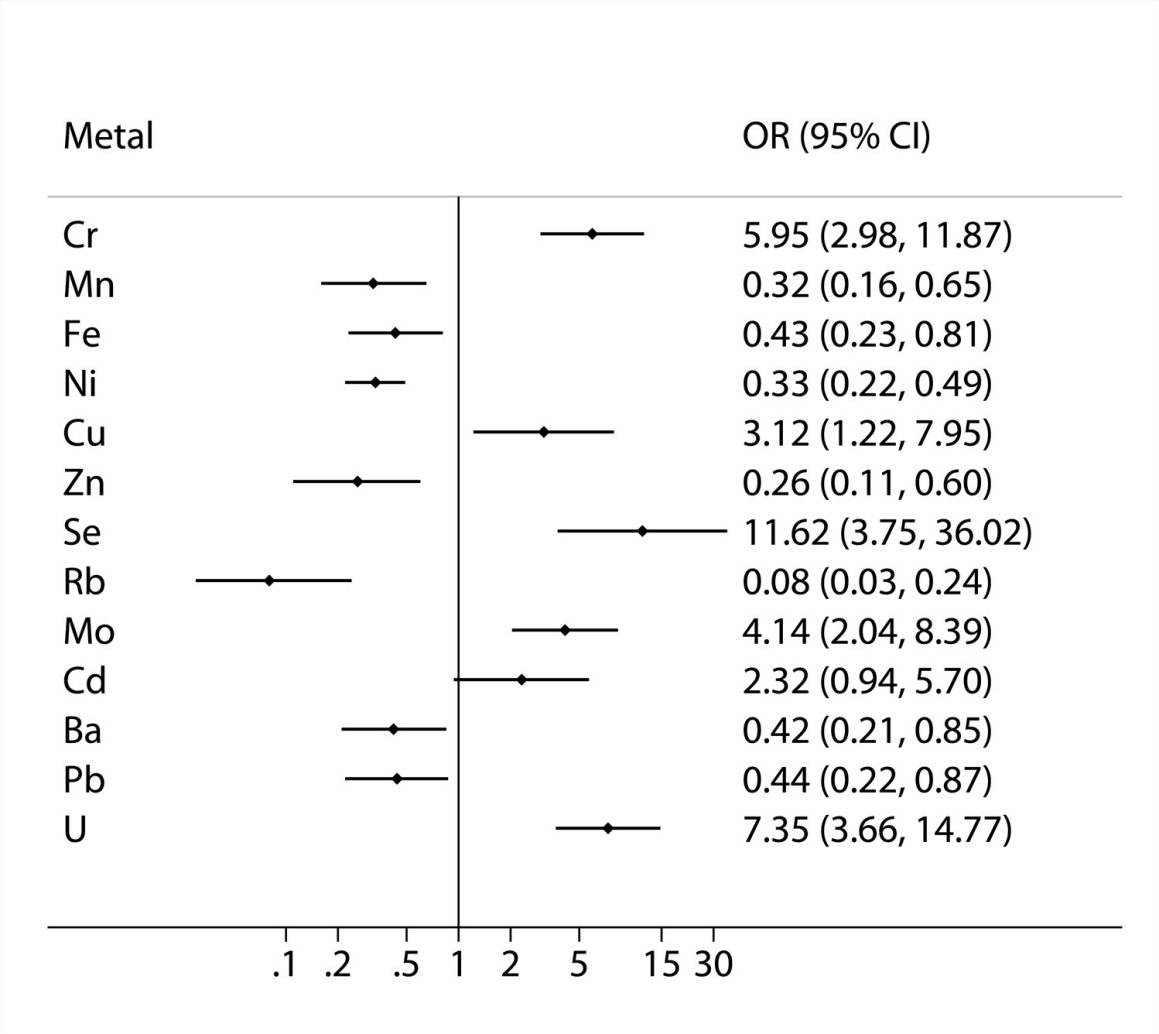
Associations between 13 metals in urine and asthma prevalence in the newly diagnosed asthma and matched controls based on the multiple-metal model. The model was adjusted for age, gender, tobacco smoking, educational level, occupational dust exposure, family history of asthma, pet ownership, flower gardening, physical activity and body mass index. Abbreviation: OR: odds ratio; CI: confidential interval.

When we performed the same analyses in the newly diagnosed asthma and the matched controls, we found that asthma prevalence was positively associated with urinary Cr, Cu, Se, Mo, Cd and U, and negatively associated with urinary Mn, Fe, Ni, Zn, Rb, Ba and Pb, in the multiple-metal models (Fig 3). When we divided asthma patients by severity (intermittent and persistent) and compared their urinary metals with those in the matched controls (Table 5), we observed similar associations of asthma with most of 13 metals. However, no significant associations were observed between 3 metals (Cd, Ba and Pb) and asthma in the group of cases with intermittent asthma and matched controls (Cd: p = 0.1976; Ba: p = 0.0549; Pb: p = 0.2394). Likewise, we didn’t find association between Fe and asthma in the group of cases with persistent asthma and matched controls (p = 0.8825).

**Table 5:** Associations between asthma and 13 selected urinary metals in different subgroups on the multiple-metal models.

Metal	Intermittent asthma and matched control		Persistent asthma and matched control	
	OR(95%CI)	p-value	OR(95%CI)	p-value
**Cr**	6.28 (2.61, 15.12)	<0.0001	5.34 (2.60, 10.93)	<0.0001
**Mn**	0.40 (0.18, 0.89)	0.0247	0.11 (0.05, 0.25)	<0.0001
**Fe**	0.14 (0.06, 0.33)	<0.0001	0.95 (0.48, 1.89)	0.8825
**Ni**	0.26 (0.15, 0.43)	<0.0001	0.25 (0.15, 0.43)	<0.0001
**Cu**	9.52 (3.34, 27.16)	<0.0001	4.55 (1.59, 13.01)	0.0048
**Zn**	0.16 (0.06, 0.41)	0.0001	0.53 (0.24, 1.15)	0.1090
**Se**	19.28 (4.56, 81.52)	<0.0001	6.72 (2.10, 21.51)	0.0013
**Rb**	0.06 (0.02, 0.24)	<0.0001	0.06 (0.02, 0.18)	<0.0001
**Mo**	5.98 (2.44, 14.68)	<0.0001	3.59 (1.68, 7.68)	0.0010
**Cd**	1.82 (0.73, 4.54)	0.1976	2.88 (1.05, 7.89)	0.0394
**Ba**	0.46 (0.21, 1.02)	0.0549	0.43 (0.21, 0.87)	0.0190
**Pb**	0.58 (0.23, 1.44)	0.2394	0.38 (0.17, 0.84)	0.0159
**U**	6.56 (3.27, 13.17)	<0.0001	8.72 (4.11, 18.48)	<0.0001

Abbreviation: OR: odds ratio; CI: confidential interval.

The model was adjusted by educational level, occupational dust exposure, family history of asthma, tobacco smoking, pet ownership, flower gardening, physical activity, and body mass index.

### Correlations between urinary metals and lung function

We further investigated the correlations between concentrations of 13 asthma-related metals and lung function with adjustment for age, gender, occupational dust exposure, family history of asthma, tobacco smoking, physical activity, and BMI (Table 6). We observed the significant positive correlations between FEV_1_ and 3 urinary metals (Mn, Fe and Ni), and significant negative correlation between FEV_1_ and Cd. Additionally, the ratio of FEV_1_/FVC was positively correlated with 7 urinary metals (Mn, Fe, Ni, Zn, Rb, Ba and Pb), and negatively correlated with 5 urinary metals (Cr, Cu, Se, Mo and Cd).

**Table 6:** Correlations between 13 selected urinary metals and lung function.

Metal	FEV_1_(L)		FEV_1_/FVC (%)	
	*r (95%CI)*	p-value	*r (95%CI)*	p-value
**Cr**	0.01(-0.05, 0.07)	0.7839	-0.13(-0.18, -0.06)	<0.0001
**Mn**	0.07(0.01, 0.14)	0.0165	0.26(0.20, 0.31)	<0.0001
**Fe**	0.08(0.02, 0.14)	0.0095	0.31(0.26, 0.37)	<0.0001
**Ni**	0.08(0.02, 0.14)	0.0146	0.25(0.19, 0.31)	<0.0001
**Cu**	0.01(-0.05, 0.07)	0.714	-0.09(-0.15, -0.03)	0.0055
**Zn**	0.05(-0.01, 0.11)	0.1244	0.16(0.10, 0.22)	<0.0001
**Se**	-0.06(-0.12, 0.01)	0.0644	-0.18(-0.24, -0.12)	<0.0001
**Rb**	-0.01(-0.07, 0.05)	0.6767	0.12(0.06, 0.18)	0.0002
**Mo**	-0.04(-0.10, 0.03)	0.2564	-0.19(-0.25, -0.13)	<0.0001
**Cd**	-0.10(-0.16, -0.03)	0.0022	-0.12(-0.18, -0.06)	<0.0001
**Ba**	0.03(-0.03, 0.09)	0.2803	0.12(0.06, 0.18)	<0.0001
**Pb**	-0.01(-0.07, 0.05)	0.7126	0.16(0.10, 0.22)	<0.0001
**U**	0.01(-0.05, 0.07)	0.7213	-0.06(-0.12, 0.01)	0.0681

Abbreviation: FEV_1_, forced expiratory volume in 1 s; FVC, forced vital capacity.

Adjusted for age, gender, occupational dust exposure, family history of asthma, tobacco smoking, physical activity, and body mass index.

## Discussion

Environmental factors were commonly considered to be associated with the incidence and prevalence of asthma. Up to now, the results investigating the association between metal elements and childhood asthma were inconsistent. The research on the effects of metal elements on adult-onset asthma is rare. In the present study, we observed that the prevalence of adult asthma was positively associated with increased urinary levels of Cr, Cu, Se, Mo, Cd, and U. In contrast, levels of seven metals (Mn, Fe, Ni, Zn, Rb, Ba and Pb) were negatively associated with adult asthma.

Some previous epidemiologic studies have reported associations of exposure to the known carcinogens Cr or Cd with an increased risk of asthma [31, 32]. In this study, we also observed a positive association of Cr with asthma. In vitro and in vivo, exposure to Cr (Ⅵ) could result in inflammation of lung tissue and release of inflammatory cytokines [33, 34]. Furthermore, inhalation of particulate forms of Cr (Ⅵ) may augment the severity of ongoing allergic asthma in mice [35]. Similarly, high serum Cd concentrations have been reported to contribute to increased oxidative stress and inflammation in the lung [36, 37].

Se is an essential micronutrient for human health, being a cofactor for enzymes with antioxidant activity that protect the organism from oxidative damage. Several investigations have showed the lower levels of Se were associated with recurrent wheezing and asthma [9]. However, other studies failed to find any association between Se levels and asthma [10]. A positive association of Se intake and bronchial responsiveness was observed in young Chilean adults [38], which was in consistent with our findings. The inconclusive results might be attributed to the different sites where the studies conducted. A multi-center study of plasma Se and asthma illustrated that a 10 μg/l increase in plasma Se was associated with a 52% decrease in risk of asthma in Lodz and a 35% increase in risk in Ghent, with a 68% increase in risk in Amsterdam [19]. Wuhan is located in Jianghan plain area which is abundant in Se. The urinary Se levels among the population in Wuhan were possibly higher. Moreover, animal models have suggested that any association between Se and AHR might not be linear [39]. Although increasing Se intake reduces oxidative stress, it may also boost immune responses. Se mainly induced inflammation and AHR at low or medium levels, while Se may have antioxidant effects while inducing inflammation at high levels, resulting in an overall reduction in asthma [39]. This dose-response manner may partly explain the inconsistent results of previous studies.

Only a few studies have shown that Mo, an essential element, is involved in lung injury. Molybdenum trioxide exposed workers had higher percentage counts of lymphocytes and neutrophils in bronchoalveolar lavage [40]. An animal study observed increased incidences of alveolar/bronchiolar adenoma or carcinoma (combined) in Mo-exposed mice [41]. In the present study, we found that the high Mo levels were positively associated with prevalence of asthma. Possible mechanisms may involve the effects of Mo on respiratory inflammatory stimuli and oxidative stress [41].

Zn is a trace element and plays a key role in the regulation of immune system. Fe is an essential element and performs several crucial functions. Cu is an integral part of many important enzymes, but chronic Cu-overload and/or excess exposure will initiate oxidative damage [42]. In the current study, Zn and Fe played protective roles against asthma, but Cu appeared to be a risk factor for asthma. Several previous studies have also reported lower concentrations of Zn, higher concentrations of Cu and a higher Cu/Zn ratio in the serum of asthma cases when compared with those of controls [12, 43, 44]. Interestingly, the increase of Cu/Zn ratio was thought to be more important than their separate increases or reductions [45]. In our study, we found that the OR of Cu/Zn reached 3.87 (3.07, 4.86) in the single-metal model which was higher than that of Cu. In addition, a nutritional supplement study showed that after receiving daily multiple nutrient supplements for two months, asthmatics had markedly reduced Cu and malondialdehyde levels, increased Zn levels and alleviation of syndromes [46]. It was postulated that an increase in serum Cu might lead to diminishment of serum Zn and could thus implicitly cause inflammation by decreasing the capability of the antioxidant system [12]. Similarly, Fe supplementation markedly decreased allergen-induced AHR, eosinophil infiltration, and production of pro-inflammatory cytokines [47]. Moreover, Zn exposure increased Fe uptake in respiratory epithelial cells and subsequently increased the expression of divalent metal transporter 1 and ferritin, which could diminish oxidative stress [48].

Mn is an essential element involved in antioxidant activities. In accordance with our results, Patel et al. found that symptomatic asthma in adults was associated with low dietary intake of Mn [49], and the lowest intakes of Mn were associated with a more than 5 times higher risk of bronchial reactivity [50]. Several studies also revealed that Mn-related antioxidants could mitigate the effects of oxidative stress in an asthma mice model, and a Mn -porphyrin compound could suppress both epithelial thickening and mucus accumulation in the epithelium [51, 52].

Due to its effects on promoting Th2 immune responses [53], Pb has been hypothesized to be a risk factor for asthma [54]. However, epidemiological evidence is controversial. Among African Americans, Joseph et al didn’t observe an effect of blood Pb levels on asthma risk [55]. In a retrospective cohort study, blood lead ≥10 μg/dL was not associated with asthma (adjusted OR = 0.91, 95% CI: 0.55, 1.48), nor was chronic lead exposure (adjusted OR = 0.95, 95% CI: 0.58, 1.55) [56]. In the present study, we even found a negative association between urinary Pb and asthma. Because Pb exposure and asthma share risk factors that are heavily influenced by socioeconomic status, it is difficult to obtain an unbiased estimate of true relationship between Pb exposure and prevalence of asthma, and levels of Pb in body may be less a predictor of asthma [55].

Some previous studies have reported adverse effects of U exposure on the respiratory system. To our knowledge, only one epidemiological study has observed a positive quantitative association between urinary U levels and asthma in adults [57], in agreement with our findings. In a birth-cohort study from New York City, a positive association was found between Ni and wheeze during the first 2 years of life [58]. Very recently, the prospective Prevention and Incidence of Asthma and Mite Allergy (PIAMA) birth cohort study found no association of Ni in particulate matter with incidence and prevalence of asthma in schoolchildren[11]. Even in our study, we observed negative association between urinary Ni and prevalence of asthma. In addition, our results suggested that levels of Ba and Rb were negatively associated with asthma, but the evidence is absent. Further investigations are needed to uncover underlying mechanisms.

Individuals exposed to a variety of metals, and we could not ignore the interaction between metals. After adjusting for other metals and confounding factors, there were some metals which were not associated with asthma prevalence in the multiple-metal models. As is a known lung, bladder, and skin carcinogen commonly found in drinking water [59]. Previous studies considered As as a risk factor for asthma. However, a cross-sectional study in the U.S. population demonstrated an insignificantly negative association between urinary As and prevalence of asthma [20]. In the current study, we only observed a positive association of urinary As with asthma in the single-metal model. The disputed results may be related to different body burdens of As in different regions [20]. The biological function of Sr in lung is unknown. An epidemiological study found that Sr bound to PM_2.5_ was associated with the reduction in FEF_25-75_ [60]. We only found a positive association of Sr with asthma in the single-metal model. This may be resulted from the effects of Sr on oxidative stress [61]. Epidemiological studies have reported associations between occupational asthma and metals in particulate matter, such as Al, V, Co, Sb, W and Tl [62–65]. However, the research in the general population is rare. In the present study, we did not find any associations between V, Co and asthma. In accordance with the result in the US adult population [57], we found a positive association between W and asthma prevalence in the single-metal model. Exposure to W could cause a marked inflammatory response in lung tissue and that the leukocyte exudates may invade alveolar areas of the lung [66]. As a continual variable, a positive association of borderline significance was observed for Sn in the single-metal model. But we did not observed a significant association of Sn with asthma, when dividing urinary concentrations of Sn into quartiles. The possible reason may be that the urinary concentrations of Sn were lower than LOQ in 26.50% of the samples and were replaced with half the LOQ.

The exact pathogenesis of asthma remains unclear, but airway inflammation and oxidative stress are believed to play crucial roles. They are also considered as the main pathways through which metals induce and influence the development of asthma. For instance, heavy metals, such as Cd and Cr, could induce airway inflammatory responses [35, 36], and lead to AHR and airway obstruction [67]. Activated immune cells undergo a respiratory burst with the generation of oxidants, such as reactive oxygen species, which are reported to sensitize airway muscles to acetylcholine-induced contraction [68], induce AHR [69], and increase mucus secretion and epithelial shedding [70]. Some metals like Se, Zn and Mn can also influence the oxidant/antioxidant balance, and further regulate inflammatory responses [71]. The association between inflammation and oxidative stress could set up a positive-feedback loop that exacerbates asthma.

Our study has several strengths. First, multiple logistic regression analysis allowed us to control for potential confounding factors which are supposed to be related with asthma. Second, we simultaneously determined a wide range of metals in urine to evaluate personal exposure from all sources, and established dose-response relationships between urinary metals and adult asthma. Third, newly diagnosed asthma and asthma of different severity were compared with the matched controls, and the associations of urinary metals with asthma were nearly the same. Several limitations in this study should be considered. First, one time urine samples were used for analysis, and might result in measurement error due to individual variability in short-term metal excretion and influenced the significance of the findings. However, it is not practical to collect 24-h urine samples in the large population study. Second, some asthma cases had been diagnosed several years previously. Therefore, they may have adjusted living habits following doctors’ recommendations, resulting in that incidence of smoking and flower gardening among asthma cases were not higher than those among the controls. Third, the limitation of hospital based case-control study is that we missed some asymptomatic cases because they don’t attend in the hospital. Forth, although we observed associations between urinary metal concentrations and adult asthma, the case-control study remains difficult to establish a causal relationship. Prospective studies are warranted to establish the causal relationships between metal exposure and asthma.

## Conclusions

The findings of this study suggest that asthma prevalence in the Chinese adults was positively associated with urinary Cr, Cu, Se, Mo, Cd, and U, and negatively associated with urinary Mn, Fe, Ni, Zn, Rb, Ba and Pb. The results are partly in accordance with previous studies, and the mechanisms of some metals remain unknown. Given that exposure to metals differs by regions, as well as the possible error due to Serial measurements of a variety of metals, our preliminary findings need to be replicated in large populations in different regions.
